# Ethyl 6-(4-meth­oxy­phen­yl)-2-oxo-4-phenyl­cyclo­hex-3-ene­carboxyl­ate

**DOI:** 10.1107/S1600536812036446

**Published:** 2012-08-25

**Authors:** Hoong-Kun Fun, Abbas Farhadikoutenaei, B. K. Sarojini, B. J. Mohan, B. Narayana

**Affiliations:** a X-ray Crystallography Unit, School of Physics, Universiti Sains Malaysia, 11800 USM, Penang, Malaysia; b Department of Chemistry, P.A. College of Engineering, Mangalore 574 153, India; c Department of Chemistry, Mangalore University, Mangalagangotri 574 199, Mangalore, India

## Abstract

The asymmetric unit of the title compound, C_22_H_22_O_4_, consists of two independent mol­ecules (*A* and *B*). The cyclo­hexene rings adopt slightly distorted sofa conformations in both mol­ecules. The dihedral angles between the benzene rings are 74.16 (13) and 71.85 (13)° in mol­ecules *A* and *B*, respectively. In the crystal, weak C—H⋯O hydrogen bonds link the mol­ecules into a ribbon-like structure along the *b* axis. Weak C—H⋯π inter­actions are also observed.

## Related literature

For applications of chalcones and cyclo­hexenone derivatives, see: Padmavathi *et al.* (2000 ▶); Senguttuvan & Nagarajan (2010 ▶); Tanaka *et al.* (1997 ▶). For related structures, see: Dutkiewicz *et al.* (2011*a* ▶,*b* ▶,*c* ▶); Fun *et al.* (2008 ▶); Fischer *et al.* (2008 ▶). For conformation analysis, see: Cremer & Pople (1975 ▶). For bond-length data, see: Allen *et al.* (1987 ▶). For the stability of the temperature controller used in the data collection, see: Cosier & Glazer (1986 ▶).
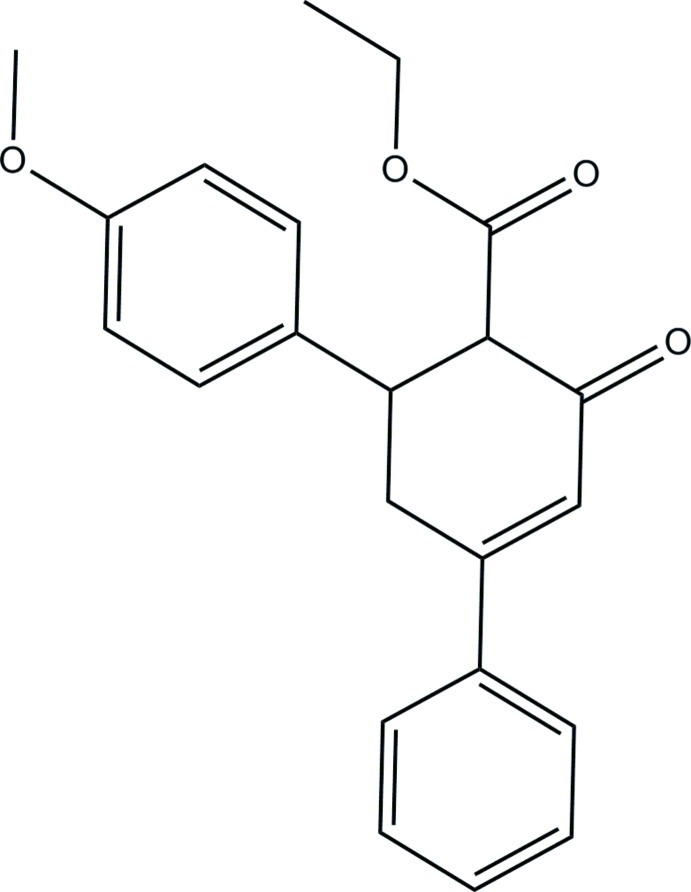


## Experimental

### 

#### Crystal data

C_22_H_22_O_4_
*M* *_r_* = 350.40
Orthorhombic, 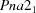

*a* = 22.3528 (13) Å
*b* = 8.1659 (5) Å
*c* = 19.7132 (12) Å
*V* = 3598.3 (4) Å^3^
*Z* = 8
Mo *K*α radiation
μ = 0.09 mm^−1^
*T* = 100 K
0.37 × 0.24 × 0.17 mm

#### Data collection

Bruker APEX DUO CCD area-detector diffractometer
Absorption correction: multi-scan (*SADABS*; Bruker, 2009 ▶) *T*_min_ = 0.968, *T*_max_ = 0.985
35140 measured reflections
5622 independent reflections
4938 reflections with *I* > 2σ(*I*)
*R*_int_ = 0.052

#### Refinement

*R*[*F* ^2^ > 2σ(*F* ^2^)] = 0.049
*wR*(*F* ^2^) = 0.139
*S* = 1.08
5622 reflections
473 parameters
1 restraint
H-atom parameters constrained
Δρ_max_ = 0.34 e Å^−3^
Δρ_min_ = −0.24 e Å^−3^

### 

Data collection: *APEX2* (Bruker, 2009 ▶); cell refinement: *SAINT* (Bruker, 2009 ▶); data reduction: *SAINT*; program(s) used to solve structure: *SHELXTL* (Sheldrick, 2008 ▶); program(s) used to refine structure: *SHELXTL*; molecular graphics: *SHELXTL*; software used to prepare material for publication: *SHELXTL* and *PLATON* (Spek, 2009 ▶).

## Supplementary Material

Crystal structure: contains datablock(s) globl, I. DOI: 10.1107/S1600536812036446/lh5518sup1.cif

Structure factors: contains datablock(s) I. DOI: 10.1107/S1600536812036446/lh5518Isup2.hkl

Supplementary material file. DOI: 10.1107/S1600536812036446/lh5518Isup3.cml

Additional supplementary materials:  crystallographic information; 3D view; checkCIF report

## Figures and Tables

**Table 1:** Hydrogen-bond geometry (Å, °) *Cg*1 and *Cg*2 are the centroids of the C13*A*–C18*A* and C13*B*–C18*B* rings, respectively.

*D*—H⋯*A*	*D*—H	H⋯*A*	*D*⋯*A*	*D*—H⋯*A*
C5*A*—H5*AA*⋯O1*A* ^i^	0.95	2.41	3.210 (3)	141
C5*B*—H5*BA*⋯O1*B* ^i^	0.95	2.53	3.329 (3)	142
C14*B*—H14*B*⋯O2*A*	0.95	2.43	3.377 (3)	173
C17*A*—H17*A*⋯O2*B* ^i^	0.95	2.59	3.218 (4)	124
C21*A*—H21*A*⋯*Cg*1^i^	0.99	2.70	3.513 (3)	142
C21*B*—H21*D*⋯*Cg*2^i^	0.99	2.71	3.501 (3)	139

Symmetry code: (i) 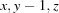.

**Fig. 1.**
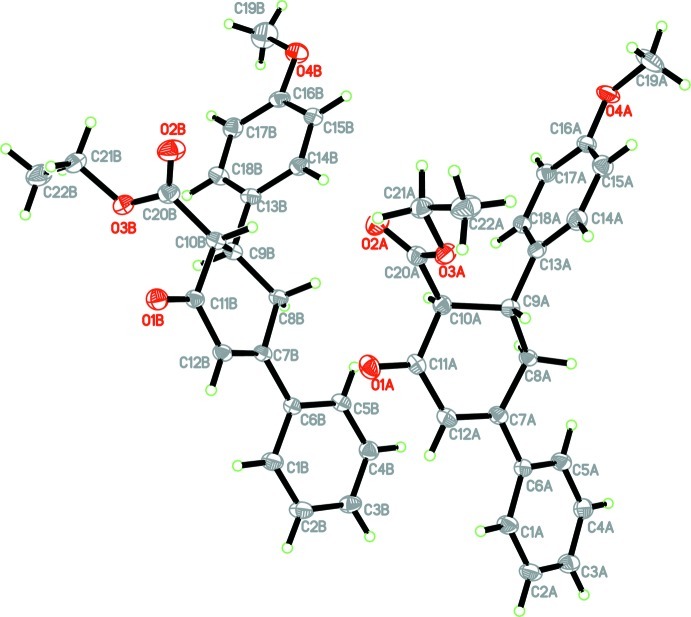
The molecular structure of the title compound, showing 50% probability displacement ellipsoids and the atom-numbering scheme.

**Fig. 2.**
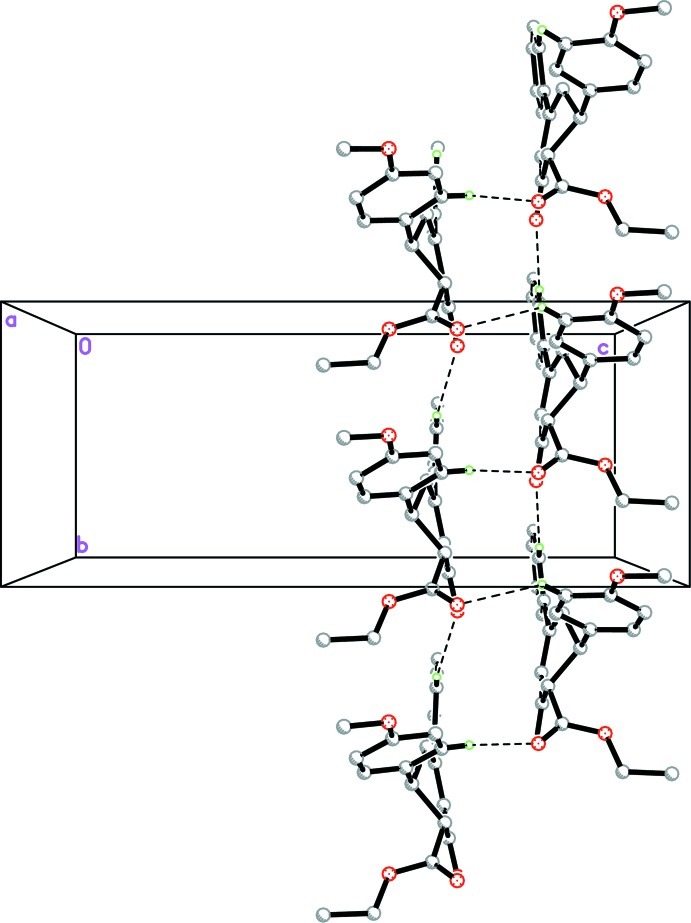
The crystal packing of the title compound, viewed along the a axis.

## supplementary crystallographic information

### Comment

Chalcones undergo a variety of chemical reactions and were found to be useful in the synthesis of various heterocyclic compounds. Michael addition of ethyl acetoacetate to chalcones yield 4,6-diaryl-2-oxo-cyclohex-3-ene-1-carboxylate derivatives, which could be used for the synthesis of fused heterocycles like isoxazoles, pyrazoles and quinazolins (Padmavathi *et al.*, 2000; Senguttuvan & Nagarajan, 2010). Cyclohexenone derivatives are well known lead molecules for the treatment of inflammation and autoimmune diseases (Tanaka *et al.*, 1997). The crystal structure of some of the cyclohexenone derivatives have been reported (Dutkiewicz *et al.*, 2011*a*,*b*,*c*; Fun *et al.*, 2008; Fischer *et al.*, 2008). The present work describes the synthesis and crystal structure of the title compound which was prepared by the reaction of 1-phenyl-3-(4-methoxyphenyl)- prop-2-en-1-one with ethyl acetoacetate.

The asymmetric unit of the title compound, (Fig 1), consists of two independent molecules (A and B). The cyclohexene rings (C7A–C12A and C7B–C12B) adopt slightly distorted sofa conformations with puckering parameters (Cremer & Pople, 1975) Q = 0.495 (3) Å, Θ = 126.1 (3) Å, φ = 316.5 (4)° and Q = 0.491 (3) Å, Θ = 54.2 (3) Å, φ = 129.5 (4)°, respectively. The dihedral angle between the benzene rings (C1–C6 and C13–C18) are 74.16 (13)° in molecule A and 71.85 (13) ° in molecule B. In the crystal (Fig 2), intermolecular C—H···O hydrogen bonds link the molecules into a ribbon-like structure along the *b* axis. The crystal structure is further stabilized by weak C—H···π interactions (Table 1), involving the C13A–C18A ring (centroid *Cg*1) and C13B–C18B ring (centroid *Cg*2).

### Experimental

1-Phenyl-3-(4-methoxyphenyl)-prop-2-en-1-one (2.38 g, 0.01 mol) and ethyl acetoacetate (1.30 g, 0.01 mol) were refluxed for 8–10 hrs in 30 ml methanol in presence of 0.8 ml of 10% NaOH. The reaction mixture was cooled to room temperature and the precipitate obtained was filtered. The single crystals were grown by slow evaporation from solvent ethanol. M.P = 375–377 K

### Refinement

All H atoms were positioned geometrically and refined using a riding model with *U*_iso_(H) = 1.2 or 1.5 *U*_eq_(C) (C—H = 0.95, 0.98, 0.99 and 1.00 Å). In the final refinement, three outliers reflections (9 2 0), (6 2 8) and (10 2 2) were omitted. A total of 5191 Friedel pairs were merged as there is no significant anomalous dispersion to determine the absolute structure.

### Crystal data

**Table:**

C_22_H_22_O_4_	*F*(000) = 1488
*M**_r_* = 350.40	*D*_x_ = 1.294 Mg m^−^^3^
Orthorhombic, *P**n**a*2_1_	Mo *K*α radiation, λ = 0.71073 Å
Hall symbol: P 2c -2n	Cell parameters from 8949 reflections
*a* = 22.3528 (13) Å	θ = 2.8–30.4°
*b* = 8.1659 (5) Å	µ = 0.09 mm^−^^1^
*c* = 19.7132 (12) Å	*T* = 100 K
*V* = 3598.3 (4) Å^3^	Block, colourles
*Z* = 8	0.37 × 0.24 × 0.17 mm

### Data collection

**Table:**

Bruker APEX DUO CCD area-detector diffractometer	5622 independent reflections
Radiation source: fine-focus sealed tube	4938 reflections with *I* > 2σ(*I*)
Graphite monochromator	*R*_int_ = 0.052
φ and ω scans	θ_max_ = 30.5°, θ_min_ = 1.8°
Absorption correction: multi-scan (*SADABS*; Bruker, 2009)	*h* = −31→31
*T*_min_ = 0.968, *T*_max_ = 0.985	*k* = −11→11
35140 measured reflections	*l* = −28→28

### Refinement

**Table:**

Refinement on *F*^2^	Primary atom site location: structure-invariant direct methods
Least-squares matrix: full	Secondary atom site location: difference Fourier map
*R*[*F*^2^ > 2σ(*F*^2^)] = 0.049	Hydrogen site location: inferred from neighbouring sites
*wR*(*F*^2^) = 0.139	H-atom parameters constrained
*S* = 1.08	*w* = 1/[σ^2^(*F*_o_^2^) + (0.0809*P*)^2^ + 0.8699*P*] where *P* = (*F*_o_^2^ + 2*F*_c_^2^)/3
5622 reflections	(Δ/σ)_max_ < 0.001
473 parameters	Δρ_max_ = 0.34 e Å^−^^3^
1 restraint	Δρ_min_ = −0.24 e Å^−^^3^

### Special details

**Table:**

Experimental. The crystal was placed in the cold stream of an Oxford Cryosystems Cobra open-flow nitrogen cryostat (Cosier & Glazer, 1986) operating at 100.0 (1) K.
Geometry. All e.s.d.'s (except the e.s.d. in the dihedral angle between two l.s. planes) are estimated using the full covariance matrix. The cell e.s.d.'s are taken into account individually in the estimation of e.s.d.'s in distances, angles and torsion angles; correlations between e.s.d.'s in cell parameters are only used when they are defined by crystal symmetry. An approximate (isotropic) treatment of cell e.s.d.'s is used for estimating e.s.d.'s involving l.s. planes.
Refinement. Refinement of *F*^2^ against ALL reflections. The weighted *R*-factor *wR* and goodness of fit *S* are based on *F*^2^, conventional *R*-factors *R* are based on *F*, with *F* set to zero for negative *F*^2^. The threshold expression of *F*^2^ > σ(*F*^2^) is used only for calculating *R*-factors(gt) *etc*. and is not relevant to the choice of reflections for refinement. *R*-factors based on *F*^2^ are statistically about twice as large as those based on *F*, and *R*- factors based on ALL data will be even larger.

### Fractional atomic coordinates and isotropic or equivalent isotropic displacement parameters (Å^2^)

**Table:**

	*x*	*y*	*z*	*U*_iso_*/*U*_eq_	
O1A	0.33599 (9)	0.6380 (2)	0.17062 (11)	0.0253 (4)	
O2A	0.19099 (10)	0.6033 (3)	0.17833 (11)	0.0286 (4)	
O3A	0.22756 (8)	0.5761 (2)	0.07262 (10)	0.0224 (4)	
O4A	0.04435 (8)	−0.0315 (2)	0.07373 (12)	0.0269 (4)	
C1A	0.49181 (11)	0.1749 (3)	0.14792 (15)	0.0229 (5)	
H1AA	0.4978	0.2892	0.1418	0.028*	
C2A	0.54107 (11)	0.0714 (3)	0.15406 (16)	0.0257 (5)	
H2AA	0.5803	0.1160	0.1531	0.031*	
C3A	0.53336 (12)	−0.0960 (3)	0.16158 (16)	0.0247 (5)	
H3AA	0.5671	−0.1663	0.1650	0.030*	
C4A	0.47572 (11)	−0.1600 (3)	0.16412 (15)	0.0230 (5)	
H4AA	0.4701	−0.2746	0.1693	0.028*	
C5A	0.42624 (11)	−0.0575 (3)	0.15908 (14)	0.0212 (5)	
H5AA	0.3872	−0.1029	0.1613	0.025*	
C6A	0.43327 (11)	0.1118 (3)	0.15070 (13)	0.0190 (5)	
C7A	0.37995 (11)	0.2194 (3)	0.14599 (13)	0.0182 (5)	
C8A	0.32006 (10)	0.1426 (3)	0.13001 (13)	0.0173 (4)	
H8AA	0.3053	0.0854	0.1710	0.021*	
H8AB	0.3257	0.0595	0.0940	0.021*	
C9A	0.27230 (10)	0.2658 (3)	0.10665 (13)	0.0189 (4)	
H9AA	0.2851	0.3106	0.0617	0.023*	
C10A	0.27010 (11)	0.4090 (3)	0.15723 (14)	0.0186 (5)	
H10A	0.2597	0.3642	0.2029	0.022*	
C11A	0.33127 (11)	0.4900 (3)	0.16184 (13)	0.0189 (4)	
C12A	0.38382 (11)	0.3828 (3)	0.15857 (13)	0.0202 (5)	
H12A	0.4222	0.4296	0.1655	0.024*	
C13A	0.21137 (10)	0.1869 (3)	0.09738 (13)	0.0188 (5)	
C14A	0.18482 (12)	0.1828 (3)	0.03368 (14)	0.0228 (5)	
H14A	0.2051	0.2305	−0.0038	0.027*	
C15A	0.12898 (11)	0.1104 (3)	0.02339 (14)	0.0229 (5)	
H15A	0.1117	0.1075	−0.0206	0.027*	
C16A	0.09903 (11)	0.0428 (3)	0.07818 (14)	0.0198 (5)	
C17A	0.12397 (11)	0.0491 (3)	0.14289 (14)	0.0196 (5)	
H17A	0.1029	0.0044	0.1805	0.024*	
C18A	0.17959 (10)	0.1207 (3)	0.15224 (13)	0.0186 (4)	
H18A	0.1964	0.1251	0.1965	0.022*	
C19A	0.01679 (15)	−0.0360 (5)	0.0087 (2)	0.0410 (8)	
H19A	−0.0222	−0.0902	0.0122	0.061*	
H19B	0.0423	−0.0971	−0.0229	0.061*	
H19C	0.0113	0.0759	−0.0081	0.061*	
C20A	0.22468 (11)	0.5390 (3)	0.13910 (14)	0.0211 (5)	
C21A	0.18806 (12)	0.7066 (3)	0.04974 (14)	0.0238 (5)	
H21A	0.1999	0.8121	0.0705	0.029*	
H21B	0.1462	0.6824	0.0627	0.029*	
C22A	0.19371 (14)	0.7157 (5)	−0.02593 (16)	0.0344 (7)	
H22A	0.1648	0.7949	−0.0437	0.052*	
H22B	0.1857	0.6076	−0.0456	0.052*	
H22C	0.2343	0.7504	−0.0380	0.052*	
O1B	0.27371 (8)	1.1276 (2)	0.29846 (11)	0.0228 (4)	
O2B	0.12384 (9)	1.0816 (3)	0.30559 (11)	0.0311 (5)	
O3B	0.17048 (8)	1.0763 (2)	0.40694 (11)	0.0236 (4)	
O4B	−0.01709 (9)	0.4697 (3)	0.41096 (12)	0.0291 (4)	
C1B	0.43083 (11)	0.6756 (3)	0.33072 (15)	0.0229 (5)	
H1BA	0.4357	0.7910	0.3332	0.028*	
C2B	0.48104 (11)	0.5751 (3)	0.32730 (17)	0.0258 (6)	
H2BA	0.5199	0.6221	0.3279	0.031*	
C3B	0.47452 (11)	0.4060 (3)	0.32307 (15)	0.0235 (5)	
H3BA	0.5088	0.3377	0.3198	0.028*	
C4B	0.41756 (11)	0.3374 (3)	0.32369 (14)	0.0221 (5)	
H4BA	0.4130	0.2219	0.3214	0.027*	
C5B	0.36740 (10)	0.4366 (3)	0.32766 (13)	0.0184 (4)	
H5BA	0.3288	0.3882	0.3284	0.022*	
C6B	0.37288 (10)	0.6080 (3)	0.33057 (13)	0.0166 (4)	
C7B	0.31931 (10)	0.7145 (3)	0.33191 (12)	0.0165 (4)	
C8B	0.25930 (10)	0.6353 (3)	0.34695 (13)	0.0182 (4)	
H8BA	0.2648	0.5503	0.3822	0.022*	
H8BB	0.2445	0.5806	0.3054	0.022*	
C9B	0.21188 (11)	0.7588 (3)	0.37141 (13)	0.0188 (4)	
H9BA	0.2255	0.8038	0.4160	0.023*	
C10B	0.20819 (10)	0.9020 (3)	0.32083 (13)	0.0186 (4)	
H10B	0.1959	0.8568	0.2758	0.022*	
C11B	0.26971 (10)	0.9824 (3)	0.31238 (13)	0.0183 (4)	
C12B	0.32191 (10)	0.8759 (3)	0.31791 (14)	0.0187 (5)	
H12B	0.3602	0.9234	0.3112	0.022*	
C13B	0.15086 (10)	0.6802 (3)	0.38198 (13)	0.0188 (5)	
C14B	0.12015 (11)	0.6010 (3)	0.32961 (14)	0.0200 (5)	
H14B	0.1380	0.5936	0.2860	0.024*	
C15B	0.06373 (11)	0.5328 (3)	0.34039 (14)	0.0200 (5)	
H15B	0.0431	0.4801	0.3043	0.024*	
C16B	0.03806 (11)	0.5427 (3)	0.40436 (15)	0.0208 (5)	
C17B	0.06745 (12)	0.6203 (3)	0.45726 (14)	0.0241 (5)	
H17B	0.0496	0.6270	0.5009	0.029*	
C18B	0.12387 (11)	0.6886 (3)	0.44514 (14)	0.0230 (5)	
H18B	0.1442	0.7421	0.4812	0.028*	
C19B	−0.04459 (16)	0.4778 (5)	0.4755 (2)	0.0444 (9)	
H19D	−0.0817	0.4141	0.4749	0.067*	
H19E	−0.0536	0.5921	0.4865	0.067*	
H19F	−0.0174	0.4327	0.5097	0.067*	
C20B	0.16238 (11)	1.0292 (3)	0.34184 (14)	0.0225 (5)	
C21B	0.12930 (12)	1.2019 (4)	0.43107 (15)	0.0251 (5)	
H21C	0.0876	1.1728	0.4190	0.030*	
H21D	0.1390	1.3089	0.4101	0.030*	
C22B	0.13615 (14)	1.2114 (5)	0.50673 (17)	0.0370 (7)	
H22D	0.1102	1.2981	0.5246	0.056*	
H22E	0.1779	1.2360	0.5180	0.056*	
H22F	0.1248	1.1063	0.5270	0.056*	

### Atomic displacement parameters (Å^2^)

**Table:**

	*U*^11^	*U*^22^	*U*^33^	*U*^12^	*U*^13^	*U*^23^
O1A	0.0282 (9)	0.0172 (8)	0.0304 (10)	−0.0021 (7)	−0.0058 (8)	−0.0003 (7)
O2A	0.0300 (10)	0.0309 (10)	0.0248 (10)	0.0055 (8)	0.0054 (8)	−0.0013 (8)
O3A	0.0206 (8)	0.0238 (9)	0.0228 (9)	0.0049 (7)	0.0015 (7)	−0.0017 (7)
O4A	0.0161 (8)	0.0289 (10)	0.0357 (11)	−0.0042 (7)	−0.0067 (8)	−0.0032 (9)
C1A	0.0182 (10)	0.0191 (11)	0.0314 (13)	−0.0025 (8)	−0.0008 (10)	0.0006 (10)
C2A	0.0162 (10)	0.0256 (13)	0.0355 (15)	−0.0033 (9)	−0.0013 (11)	−0.0006 (12)
C3A	0.0206 (10)	0.0261 (12)	0.0276 (13)	0.0023 (9)	−0.0035 (10)	0.0009 (11)
C4A	0.0235 (11)	0.0179 (11)	0.0275 (12)	0.0002 (9)	0.0006 (10)	−0.0001 (10)
C5A	0.0186 (10)	0.0230 (12)	0.0219 (12)	−0.0034 (9)	0.0007 (9)	0.0006 (10)
C6A	0.0164 (10)	0.0215 (11)	0.0191 (11)	−0.0012 (9)	0.0003 (9)	−0.0021 (9)
C7A	0.0172 (10)	0.0195 (11)	0.0178 (10)	−0.0036 (8)	0.0010 (9)	0.0017 (9)
C8A	0.0137 (9)	0.0166 (11)	0.0216 (11)	−0.0027 (8)	0.0008 (9)	−0.0005 (9)
C9A	0.0169 (10)	0.0194 (11)	0.0204 (11)	0.0000 (8)	−0.0002 (9)	−0.0012 (9)
C10A	0.0190 (10)	0.0172 (10)	0.0195 (11)	−0.0012 (8)	0.0009 (9)	−0.0010 (9)
C11A	0.0213 (10)	0.0169 (10)	0.0185 (11)	−0.0026 (8)	−0.0033 (9)	0.0003 (9)
C12A	0.0186 (10)	0.0193 (11)	0.0228 (11)	−0.0035 (8)	−0.0014 (9)	0.0007 (10)
C13A	0.0155 (9)	0.0171 (11)	0.0240 (12)	0.0007 (8)	0.0020 (9)	−0.0025 (9)
C14A	0.0230 (11)	0.0241 (12)	0.0212 (12)	−0.0016 (9)	0.0028 (9)	−0.0013 (10)
C15A	0.0234 (11)	0.0237 (12)	0.0215 (11)	0.0006 (9)	−0.0026 (10)	−0.0003 (10)
C16A	0.0162 (10)	0.0177 (11)	0.0256 (12)	−0.0001 (8)	−0.0034 (9)	−0.0014 (9)
C17A	0.0177 (10)	0.0192 (11)	0.0219 (11)	−0.0004 (8)	0.0010 (9)	0.0011 (9)
C18A	0.0179 (10)	0.0196 (11)	0.0185 (11)	0.0014 (8)	−0.0020 (9)	−0.0008 (9)
C19A	0.0316 (15)	0.0451 (19)	0.0461 (19)	−0.0067 (13)	−0.0213 (15)	−0.0034 (16)
C20A	0.0190 (10)	0.0212 (11)	0.0230 (11)	−0.0031 (9)	−0.0006 (9)	−0.0006 (10)
C21A	0.0231 (11)	0.0235 (12)	0.0248 (12)	0.0059 (10)	−0.0024 (10)	0.0004 (10)
C22A	0.0274 (14)	0.0502 (19)	0.0256 (14)	0.0096 (13)	−0.0016 (11)	0.0048 (13)
O1B	0.0211 (8)	0.0183 (8)	0.0288 (10)	−0.0020 (6)	0.0001 (7)	0.0026 (7)
O2B	0.0242 (9)	0.0453 (12)	0.0238 (9)	0.0074 (9)	−0.0008 (8)	0.0007 (9)
O3B	0.0209 (8)	0.0228 (9)	0.0269 (9)	0.0046 (7)	−0.0013 (7)	−0.0013 (8)
O4B	0.0192 (8)	0.0308 (10)	0.0374 (11)	−0.0089 (8)	0.0083 (8)	−0.0010 (9)
C1B	0.0170 (10)	0.0208 (11)	0.0309 (13)	−0.0013 (9)	−0.0009 (10)	0.0018 (10)
C2B	0.0154 (10)	0.0252 (13)	0.0368 (15)	−0.0008 (9)	0.0001 (11)	0.0046 (12)
C3B	0.0173 (10)	0.0240 (12)	0.0293 (14)	0.0036 (9)	−0.0014 (10)	0.0038 (11)
C4B	0.0208 (10)	0.0209 (11)	0.0247 (12)	−0.0002 (9)	−0.0024 (9)	0.0005 (10)
C5B	0.0179 (10)	0.0182 (10)	0.0192 (10)	−0.0026 (8)	−0.0023 (9)	0.0032 (9)
C6B	0.0151 (9)	0.0175 (10)	0.0172 (10)	−0.0011 (8)	−0.0015 (8)	0.0008 (9)
C7B	0.0144 (9)	0.0198 (11)	0.0154 (10)	−0.0015 (8)	−0.0007 (8)	0.0000 (9)
C8B	0.0140 (9)	0.0193 (11)	0.0212 (11)	−0.0016 (8)	−0.0006 (9)	0.0006 (9)
C9B	0.0165 (10)	0.0206 (11)	0.0192 (11)	−0.0021 (8)	−0.0014 (9)	0.0015 (9)
C10B	0.0156 (9)	0.0212 (11)	0.0190 (11)	−0.0016 (8)	−0.0017 (9)	0.0011 (9)
C11B	0.0160 (9)	0.0201 (11)	0.0188 (10)	−0.0021 (8)	0.0014 (8)	−0.0013 (9)
C12B	0.0139 (9)	0.0192 (11)	0.0229 (12)	−0.0036 (8)	0.0015 (9)	0.0005 (9)
C13B	0.0140 (9)	0.0195 (11)	0.0228 (11)	−0.0007 (8)	−0.0005 (9)	0.0035 (9)
C14B	0.0176 (10)	0.0215 (11)	0.0210 (11)	−0.0016 (8)	0.0041 (9)	0.0013 (9)
C15B	0.0166 (10)	0.0205 (11)	0.0228 (12)	−0.0013 (8)	0.0003 (9)	−0.0008 (9)
C16B	0.0148 (10)	0.0198 (11)	0.0278 (12)	−0.0005 (8)	0.0029 (10)	0.0037 (10)
C17B	0.0239 (11)	0.0257 (13)	0.0225 (12)	−0.0003 (10)	0.0034 (10)	0.0000 (10)
C18B	0.0199 (11)	0.0258 (12)	0.0232 (12)	−0.0036 (9)	−0.0021 (9)	0.0002 (10)
C19B	0.0331 (16)	0.0466 (19)	0.054 (2)	−0.0120 (14)	0.0238 (16)	−0.0033 (17)
C20B	0.0187 (10)	0.0235 (12)	0.0253 (12)	−0.0022 (9)	0.0039 (10)	0.0023 (10)
C21B	0.0227 (11)	0.0272 (13)	0.0256 (12)	0.0066 (10)	0.0008 (10)	0.0000 (10)
C22B	0.0293 (14)	0.055 (2)	0.0268 (14)	0.0123 (14)	−0.0036 (12)	−0.0113 (14)

### Geometric parameters (Å, º)

**Table:**

O1A—C11A	1.226 (3)	O1B—C11B	1.220 (3)
O2A—C20A	1.200 (3)	O2B—C20B	1.198 (3)
O3A—C20A	1.347 (3)	O3B—C20B	1.352 (4)
O3A—C21A	1.455 (3)	O3B—C21B	1.458 (3)
O4A—C16A	1.367 (3)	O4B—C16B	1.375 (3)
O4A—C19A	1.423 (4)	O4B—C19B	1.414 (4)
C1A—C2A	1.394 (4)	C1B—C2B	1.392 (4)
C1A—C6A	1.408 (3)	C1B—C6B	1.408 (3)
C1A—H1AA	0.9500	C1B—H1BA	0.9500
C2A—C3A	1.385 (4)	C2B—C3B	1.390 (4)
C2A—H2AA	0.9500	C2B—H2BA	0.9500
C3A—C4A	1.391 (3)	C3B—C4B	1.391 (3)
C3A—H3AA	0.9500	C3B—H3BA	0.9500
C4A—C5A	1.390 (3)	C4B—C5B	1.385 (3)
C4A—H4AA	0.9500	C4B—H4BA	0.9500
C5A—C6A	1.401 (4)	C5B—C6B	1.406 (3)
C5A—H5AA	0.9500	C5B—H5BA	0.9500
C6A—C7A	1.484 (3)	C6B—C7B	1.480 (3)
C7A—C12A	1.360 (3)	C7B—C12B	1.348 (3)
C7A—C8A	1.512 (3)	C7B—C8B	1.518 (3)
C8A—C9A	1.537 (3)	C8B—C9B	1.540 (4)
C8A—H8AA	0.9900	C8B—H8BA	0.9900
C8A—H8AB	0.9900	C8B—H8BB	0.9900
C9A—C13A	1.517 (3)	C9B—C13B	1.522 (3)
C9A—C10A	1.537 (4)	C9B—C10B	1.539 (4)
C9A—H9AA	1.0000	C9B—H9BA	1.0000
C10A—C20A	1.512 (4)	C10B—C20B	1.516 (4)
C10A—C11A	1.522 (3)	C10B—C11B	1.533 (3)
C10A—H10A	1.0000	C10B—H10B	1.0000
C11A—C12A	1.466 (3)	C11B—C12B	1.460 (3)
C12A—H12A	0.9500	C12B—H12B	0.9500
C13A—C14A	1.389 (4)	C13B—C18B	1.385 (4)
C13A—C18A	1.402 (4)	C13B—C14B	1.398 (4)
C14A—C15A	1.396 (4)	C14B—C15B	1.395 (3)
C14A—H14A	0.9500	C14B—H14B	0.9500
C15A—C16A	1.386 (4)	C15B—C16B	1.388 (4)
C15A—H15A	0.9500	C15B—H15B	0.9500
C16A—C17A	1.393 (4)	C16B—C17B	1.386 (4)
C17A—C18A	1.386 (3)	C17B—C18B	1.400 (4)
C17A—H17A	0.9500	C17B—H17B	0.9500
C18A—H18A	0.9500	C18B—H18B	0.9500
C19A—H19A	0.9800	C19B—H19D	0.9800
C19A—H19B	0.9800	C19B—H19E	0.9800
C19A—H19C	0.9800	C19B—H19F	0.9800
C21A—C22A	1.499 (4)	C21B—C22B	1.501 (4)
C21A—H21A	0.9900	C21B—H21C	0.9900
C21A—H21B	0.9900	C21B—H21D	0.9900
C22A—H22A	0.9800	C22B—H22D	0.9800
C22A—H22B	0.9800	C22B—H22E	0.9800
C22A—H22C	0.9800	C22B—H22F	0.9800
			
C20A—O3A—C21A	115.9 (2)	C20B—O3B—C21B	115.2 (2)
C16A—O4A—C19A	117.2 (2)	C16B—O4B—C19B	117.0 (3)
C2A—C1A—C6A	120.6 (2)	C2B—C1B—C6B	120.7 (2)
C2A—C1A—H1AA	119.7	C2B—C1B—H1BA	119.6
C6A—C1A—H1AA	119.7	C6B—C1B—H1BA	119.6
C3A—C2A—C1A	120.6 (2)	C1B—C2B—C3B	120.2 (2)
C3A—C2A—H2AA	119.7	C1B—C2B—H2BA	119.9
C1A—C2A—H2AA	119.7	C3B—C2B—H2BA	119.9
C2A—C3A—C4A	119.3 (2)	C4B—C3B—C2B	119.7 (2)
C2A—C3A—H3AA	120.3	C4B—C3B—H3BA	120.2
C4A—C3A—H3AA	120.3	C2B—C3B—H3BA	120.2
C3A—C4A—C5A	120.5 (2)	C5B—C4B—C3B	120.4 (2)
C3A—C4A—H4AA	119.7	C5B—C4B—H4BA	119.8
C5A—C4A—H4AA	119.7	C3B—C4B—H4BA	119.8
C4A—C5A—C6A	120.9 (2)	C4B—C5B—C6B	120.9 (2)
C4A—C5A—H5AA	119.6	C4B—C5B—H5BA	119.5
C6A—C5A—H5AA	119.6	C6B—C5B—H5BA	119.5
C5A—C6A—C1A	118.1 (2)	C5B—C6B—C1B	118.0 (2)
C5A—C6A—C7A	120.1 (2)	C5B—C6B—C7B	121.0 (2)
C1A—C6A—C7A	121.8 (2)	C1B—C6B—C7B	121.0 (2)
C12A—C7A—C6A	121.3 (2)	C12B—C7B—C6B	122.4 (2)
C12A—C7A—C8A	120.1 (2)	C12B—C7B—C8B	119.6 (2)
C6A—C7A—C8A	118.6 (2)	C6B—C7B—C8B	117.9 (2)
C7A—C8A—C9A	114.0 (2)	C7B—C8B—C9B	113.0 (2)
C7A—C8A—H8AA	108.8	C7B—C8B—H8BA	109.0
C9A—C8A—H8AA	108.8	C9B—C8B—H8BA	109.0
C7A—C8A—H8AB	108.8	C7B—C8B—H8BB	109.0
C9A—C8A—H8AB	108.8	C9B—C8B—H8BB	109.0
H8AA—C8A—H8AB	107.7	H8BA—C8B—H8BB	107.8
C13A—C9A—C10A	111.9 (2)	C13B—C9B—C10B	111.2 (2)
C13A—C9A—C8A	112.4 (2)	C13B—C9B—C8B	112.5 (2)
C10A—C9A—C8A	109.0 (2)	C10B—C9B—C8B	109.4 (2)
C13A—C9A—H9AA	107.8	C13B—C9B—H9BA	107.9
C10A—C9A—H9AA	107.8	C10B—C9B—H9BA	107.9
C8A—C9A—H9AA	107.8	C8B—C9B—H9BA	107.9
C20A—C10A—C11A	108.2 (2)	C20B—C10B—C11B	110.0 (2)
C20A—C10A—C9A	113.7 (2)	C20B—C10B—C9B	112.3 (2)
C11A—C10A—C9A	109.9 (2)	C11B—C10B—C9B	110.36 (19)
C20A—C10A—H10A	108.3	C20B—C10B—H10B	108.0
C11A—C10A—H10A	108.3	C11B—C10B—H10B	108.0
C9A—C10A—H10A	108.3	C9B—C10B—H10B	108.0
O1A—C11A—C12A	121.7 (2)	O1B—C11B—C12B	122.5 (2)
O1A—C11A—C10A	121.0 (2)	O1B—C11B—C10B	120.4 (2)
C12A—C11A—C10A	117.2 (2)	C12B—C11B—C10B	117.0 (2)
C7A—C12A—C11A	122.9 (2)	C7B—C12B—C11B	124.3 (2)
C7A—C12A—H12A	118.6	C7B—C12B—H12B	117.8
C11A—C12A—H12A	118.6	C11B—C12B—H12B	117.8
C14A—C13A—C18A	118.1 (2)	C18B—C13B—C14B	118.2 (2)
C14A—C13A—C9A	120.2 (2)	C18B—C13B—C9B	119.5 (2)
C18A—C13A—C9A	121.7 (2)	C14B—C13B—C9B	122.3 (2)
C13A—C14A—C15A	121.6 (2)	C15B—C14B—C13B	121.1 (2)
C13A—C14A—H14A	119.2	C15B—C14B—H14B	119.5
C15A—C14A—H14A	119.2	C13B—C14B—H14B	119.5
C16A—C15A—C14A	119.2 (2)	C16B—C15B—C14B	119.3 (2)
C16A—C15A—H15A	120.4	C16B—C15B—H15B	120.4
C14A—C15A—H15A	120.4	C14B—C15B—H15B	120.4
O4A—C16A—C15A	124.0 (2)	O4B—C16B—C17B	123.5 (2)
O4A—C16A—C17A	115.7 (2)	O4B—C16B—C15B	115.5 (2)
C15A—C16A—C17A	120.4 (2)	C17B—C16B—C15B	121.0 (2)
C18A—C17A—C16A	119.7 (2)	C16B—C17B—C18B	118.8 (2)
C18A—C17A—H17A	120.1	C16B—C17B—H17B	120.6
C16A—C17A—H17A	120.1	C18B—C17B—H17B	120.6
C17A—C18A—C13A	121.0 (2)	C13B—C18B—C17B	121.7 (2)
C17A—C18A—H18A	119.5	C13B—C18B—H18B	119.1
C13A—C18A—H18A	119.5	C17B—C18B—H18B	119.1
O4A—C19A—H19A	109.5	O4B—C19B—H19D	109.5
O4A—C19A—H19B	109.5	O4B—C19B—H19E	109.5
H19A—C19A—H19B	109.5	H19D—C19B—H19E	109.5
O4A—C19A—H19C	109.5	O4B—C19B—H19F	109.5
H19A—C19A—H19C	109.5	H19D—C19B—H19F	109.5
H19B—C19A—H19C	109.5	H19E—C19B—H19F	109.5
O2A—C20A—O3A	123.9 (3)	O2B—C20B—O3B	124.1 (3)
O2A—C20A—C10A	125.2 (3)	O2B—C20B—C10B	124.6 (3)
O3A—C20A—C10A	110.9 (2)	O3B—C20B—C10B	111.3 (2)
O3A—C21A—C22A	107.1 (2)	O3B—C21B—C22B	107.2 (2)
O3A—C21A—H21A	110.3	O3B—C21B—H21C	110.3
C22A—C21A—H21A	110.3	C22B—C21B—H21C	110.3
O3A—C21A—H21B	110.3	O3B—C21B—H21D	110.3
C22A—C21A—H21B	110.3	C22B—C21B—H21D	110.3
H21A—C21A—H21B	108.6	H21C—C21B—H21D	108.5
C21A—C22A—H22A	109.5	C21B—C22B—H22D	109.5
C21A—C22A—H22B	109.5	C21B—C22B—H22E	109.5
H22A—C22A—H22B	109.5	H22D—C22B—H22E	109.5
C21A—C22A—H22C	109.5	C21B—C22B—H22F	109.5
H22A—C22A—H22C	109.5	H22D—C22B—H22F	109.5
H22B—C22A—H22C	109.5	H22E—C22B—H22F	109.5
			
C6A—C1A—C2A—C3A	1.4 (5)	C6B—C1B—C2B—C3B	0.6 (5)
C1A—C2A—C3A—C4A	−1.1 (5)	C1B—C2B—C3B—C4B	−1.3 (5)
C2A—C3A—C4A—C5A	0.1 (5)	C2B—C3B—C4B—C5B	0.8 (4)
C3A—C4A—C5A—C6A	0.6 (4)	C3B—C4B—C5B—C6B	0.5 (4)
C4A—C5A—C6A—C1A	−0.3 (4)	C4B—C5B—C6B—C1B	−1.3 (4)
C4A—C5A—C6A—C7A	−179.5 (3)	C4B—C5B—C6B—C7B	177.4 (2)
C2A—C1A—C6A—C5A	−0.7 (4)	C2B—C1B—C6B—C5B	0.7 (4)
C2A—C1A—C6A—C7A	178.5 (3)	C2B—C1B—C6B—C7B	−178.0 (3)
C5A—C6A—C7A—C12A	159.7 (3)	C5B—C6B—C7B—C12B	−163.2 (3)
C1A—C6A—C7A—C12A	−19.5 (4)	C1B—C6B—C7B—C12B	15.4 (4)
C5A—C6A—C7A—C8A	−17.5 (4)	C5B—C6B—C7B—C8B	14.3 (4)
C1A—C6A—C7A—C8A	163.4 (3)	C1B—C6B—C7B—C8B	−167.0 (2)
C12A—C7A—C8A—C9A	19.2 (3)	C12B—C7B—C8B—C9B	−22.5 (3)
C6A—C7A—C8A—C9A	−163.6 (2)	C6B—C7B—C8B—C9B	159.9 (2)
C7A—C8A—C9A—C13A	−174.7 (2)	C7B—C8B—C9B—C13B	176.3 (2)
C7A—C8A—C9A—C10A	−50.1 (3)	C7B—C8B—C9B—C10B	52.2 (3)
C13A—C9A—C10A—C20A	−55.4 (3)	C13B—C9B—C10B—C20B	55.3 (3)
C8A—C9A—C10A—C20A	179.7 (2)	C8B—C9B—C10B—C20B	−179.8 (2)
C13A—C9A—C10A—C11A	−176.8 (2)	C13B—C9B—C10B—C11B	178.4 (2)
C8A—C9A—C10A—C11A	58.2 (3)	C8B—C9B—C10B—C11B	−56.7 (3)
C20A—C10A—C11A—O1A	20.7 (3)	C20B—C10B—C11B—O1B	−26.1 (3)
C9A—C10A—C11A—O1A	145.4 (3)	C9B—C10B—C11B—O1B	−150.5 (3)
C20A—C10A—C11A—C12A	−162.1 (2)	C20B—C10B—C11B—C12B	157.3 (2)
C9A—C10A—C11A—C12A	−37.4 (3)	C9B—C10B—C11B—C12B	32.9 (3)
C6A—C7A—C12A—C11A	−173.0 (2)	C6B—C7B—C12B—C11B	174.0 (2)
C8A—C7A—C12A—C11A	4.1 (4)	C8B—C7B—C12B—C11B	−3.5 (4)
O1A—C11A—C12A—C7A	−177.1 (3)	O1B—C11B—C12B—C7B	−178.7 (3)
C10A—C11A—C12A—C7A	5.7 (4)	C10B—C11B—C12B—C7B	−2.2 (4)
C10A—C9A—C13A—C14A	120.2 (3)	C10B—C9B—C13B—C18B	−115.0 (3)
C8A—C9A—C13A—C14A	−116.8 (3)	C8B—C9B—C13B—C18B	121.9 (3)
C10A—C9A—C13A—C18A	−58.0 (3)	C10B—C9B—C13B—C14B	63.9 (3)
C8A—C9A—C13A—C18A	65.0 (3)	C8B—C9B—C13B—C14B	−59.1 (3)
C18A—C13A—C14A—C15A	−2.1 (4)	C18B—C13B—C14B—C15B	0.3 (4)
C9A—C13A—C14A—C15A	179.6 (2)	C9B—C13B—C14B—C15B	−178.7 (2)
C13A—C14A—C15A—C16A	0.8 (4)	C13B—C14B—C15B—C16B	−0.6 (4)
C19A—O4A—C16A—C15A	−1.1 (4)	C19B—O4B—C16B—C17B	0.8 (4)
C19A—O4A—C16A—C17A	178.3 (3)	C19B—O4B—C16B—C15B	−179.9 (3)
C14A—C15A—C16A—O4A	−179.8 (2)	C14B—C15B—C16B—O4B	−178.8 (2)
C14A—C15A—C16A—C17A	0.9 (4)	C14B—C15B—C16B—C17B	0.5 (4)
O4A—C16A—C17A—C18A	179.4 (2)	O4B—C16B—C17B—C18B	179.1 (2)
C15A—C16A—C17A—C18A	−1.2 (4)	C15B—C16B—C17B—C18B	−0.2 (4)
C16A—C17A—C18A—C13A	−0.2 (4)	C14B—C13B—C18B—C17B	0.1 (4)
C14A—C13A—C18A—C17A	1.8 (4)	C9B—C13B—C18B—C17B	179.1 (2)
C9A—C13A—C18A—C17A	−180.0 (2)	C16B—C17B—C18B—C13B	−0.1 (4)
C21A—O3A—C20A—O2A	2.1 (4)	C21B—O3B—C20B—O2B	−1.6 (4)
C21A—O3A—C20A—C10A	−176.8 (2)	C21B—O3B—C20B—C10B	178.4 (2)
C11A—C10A—C20A—O2A	−101.5 (3)	C11B—C10B—C20B—O2B	107.5 (3)
C9A—C10A—C20A—O2A	136.1 (3)	C9B—C10B—C20B—O2B	−129.1 (3)
C11A—C10A—C20A—O3A	77.4 (3)	C11B—C10B—C20B—O3B	−72.5 (3)
C9A—C10A—C20A—O3A	−45.0 (3)	C9B—C10B—C20B—O3B	50.8 (3)
C20A—O3A—C21A—C22A	−174.2 (2)	C20B—O3B—C21B—C22B	168.0 (2)

### Hydrogen-bond geometry (Å, º)

Cg1 and Cg2 are the centroids of the C13A–C18A and C13B–C18B rings, respectively.

**Table:**

*D*—H···*A*	*D*—H	H···*A*	*D*···*A*	*D*—H···*A*
C5*A*—H5*AA*···O1*A*^i^	0.95	2.41	3.210 (3)	141
C5*B*—H5*BA*···O1*B*^i^	0.95	2.53	3.329 (3)	142
C14*B*—H14*B*···O2*A*	0.95	2.43	3.377 (3)	173
C17*A*—H17*A*···O2*B*^i^	0.95	2.59	3.218 (4)	124
C21*A*—H21*A*···*Cg*1^i^	0.99	2.70	3.513 (3)	142
C21*B*—H21*D*···*Cg*2^i^	0.99	2.71	3.501 (3)	139

Symmetry code: (i) *x*, *y*−1, *z*.
